# Supplementary figures and images for: Young children integrate current observations, priors and agent information to predict others’ actions
Source: PLoS One. 2019 May 22;14(5):e0200976. doi: 10.1371/journal.pone.0200976 (PMC6530825; doi:10.1371/journal.pone.0200976)

## ANALYSED TRIALS

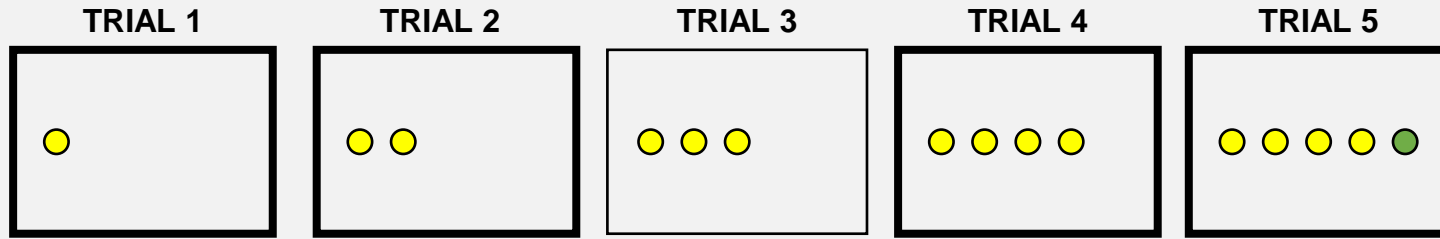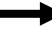

## TRIAL TIMING

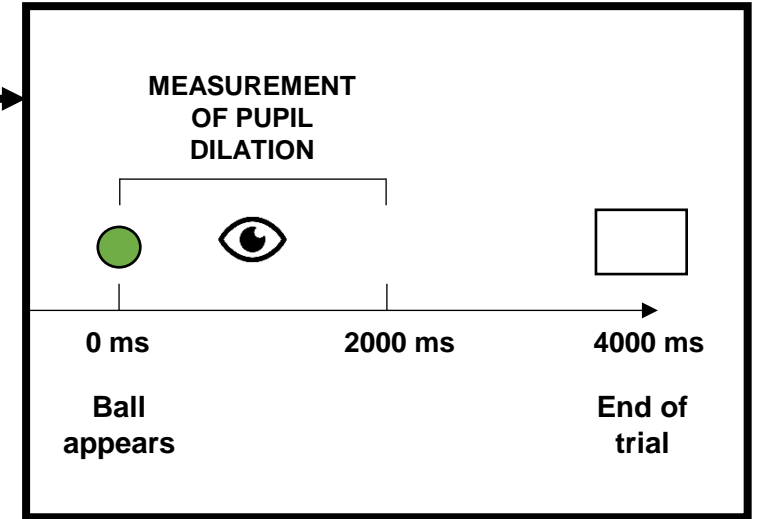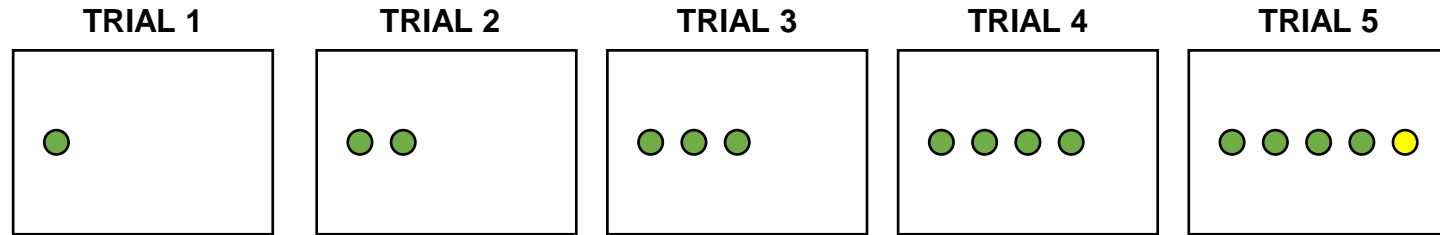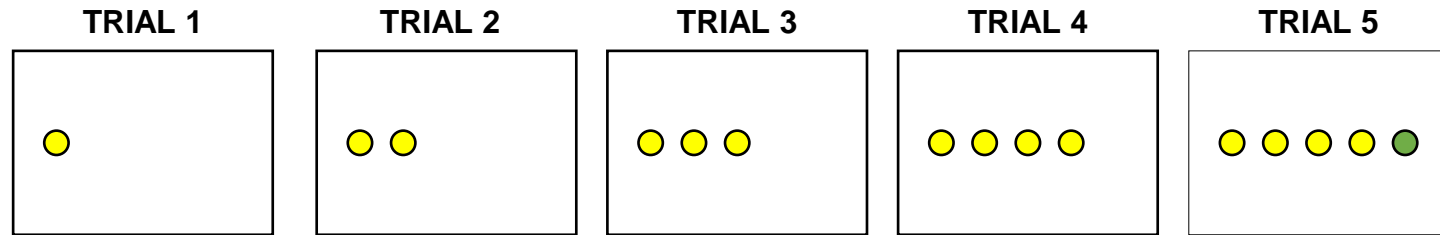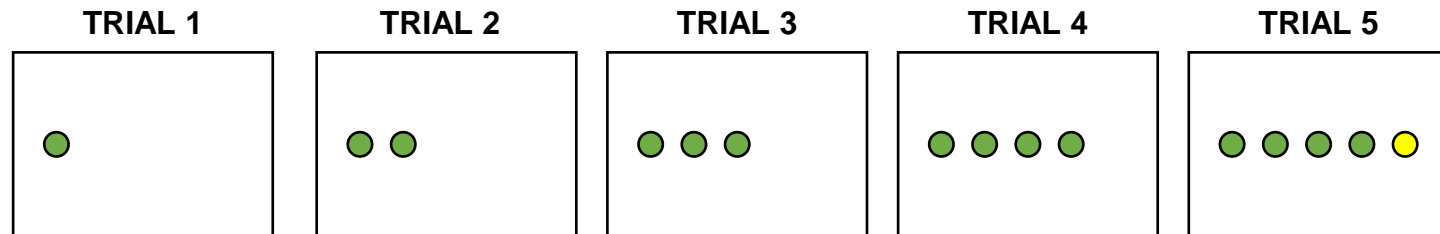

Supplement: S2 File — (PDF) [file pone.0200976.s002.pdf]
