# Supplementary material for: Young children integrate current observations, priors and agent information to predict others’ actions
Source: PLoS One. 2019 May 22;14(5):e0200976. doi: 10.1371/journal.pone.0200976 (PMC6530825; doi:10.1371/journal.pone.0200976)
Supplement: S1 Appendix — (DOCX) [file pone.0200976.s004.docx]

**Appendix**

**Computational Model**

There is an emerging literature using computational modeling to formalize how infants make inductive inferences. For example, in their 2010 study, Gweon, Tenenbaum and Schulz used a Bayesian model to describe the likelihood of different action outcomes [1]. Moreover, Jara-Ettinger et al. (2018) modeled expected behavior using a Markov Decision Process where the probability of the next action depends on the expected reward [2]. Lucas et al. (2014) modeled discrete-choice preferences using a Mixed Multinomial Logit model [3]. Taken together, these studies model the expected behavior based on some inference mechanism rather than an internal process. Here, we take a different approach and model actual surprise of the infant as hypothesized by the predictive processing paradigm under several hypothesized assumptions.

We formalized the three hypotheses described in the introduction of this paper in a set of computational models. These computational models, based on the *causal Bayesian network* formalization of predictive processing as proposed in Kwisthout, Bekkering, and van Rooij (2017), compute posterior probability distributions that represent the expectation or *prediction* of the infant prior to each ball drawn [4]. This prediction is compared to the actually observed event, yielding the prediction error. The *size* of this prediction error, quantified as the Kullback-Leibler divergence between the observed and predicted probability distributions [5], is a qualitative proxy for the pupil dilation. The prediction error is instrumental in updating the current beliefs that give rise to future predictions. These sub-processes in predictive processing (prediction, observation, prediction error computation, and belief updating) are graphically depicted in the context of the experimental paradigm in Fig A.

**Fig A:** Sub-processes in predictive processing: prediction, observation, prediction error computation, and belief updating. Green and yellow bars represent probability distributions.


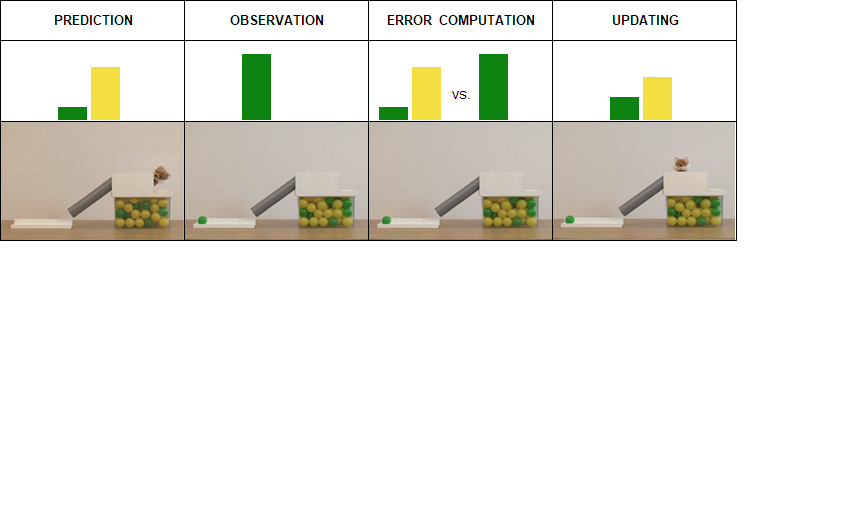


In the predictive processing account, the brain continuously predicts its inputs using generative models that represent the causal structure of the world [6]. In our computational characterization, these generative models take the form of causal Bayesian networks [7] with hypothesis nodes (representing the potential causes of the phenomena observed), prediction nodes (representing the observable information), and intermediate nodes (representing contextual information). The attributed *preference* of the agent is represented as a hypothesis node, the predicted *outcome* of the draw is represented as a prediction node, and the *container distribution* and the *previous draws* are represented as intermediate nodes. In the model representing Hypothesis 1, the container distribution and the preference of the agent is absent, and predictions are solely made based on the previous balls drawn. In the model representing Hypothesis 2, the container distribution is included, but the preference of the agent is still absent. In the model representing Hypothesis 3, finally, all three aspects are included. Fig B graphically depicts the structure of these three models prior to drawing one of the balls.

**Fig B:** In the left panel, the model for H1 is depicted. Preceding every ball that is drawn from the box, the prediction (represented by the prediction variable Pred) is based on the previous ball (intermediate variable Prev) only; a uniform distribution is assumed for the first ball. There are no hypotheses regarding picking bias; also, the content of the container is not modeled as it is ignored in H1. The model for H2 is depicted in the middle panel. Here, in addition to the information regarding the previous ball, the contents of the container form a contextual influence that modulates the prediction; here modeled as an additional intermediate variable Box distribution. In the right panel, the model for H3 is depicted, in which in addition to the available contextual information also the attributed agent preference is included. As this preference is a causal explanation for the ball drawn – rather than a contextual influence – which can be updated in the light of prediction errors, we model this variable as a hypothesis variable Hyp.


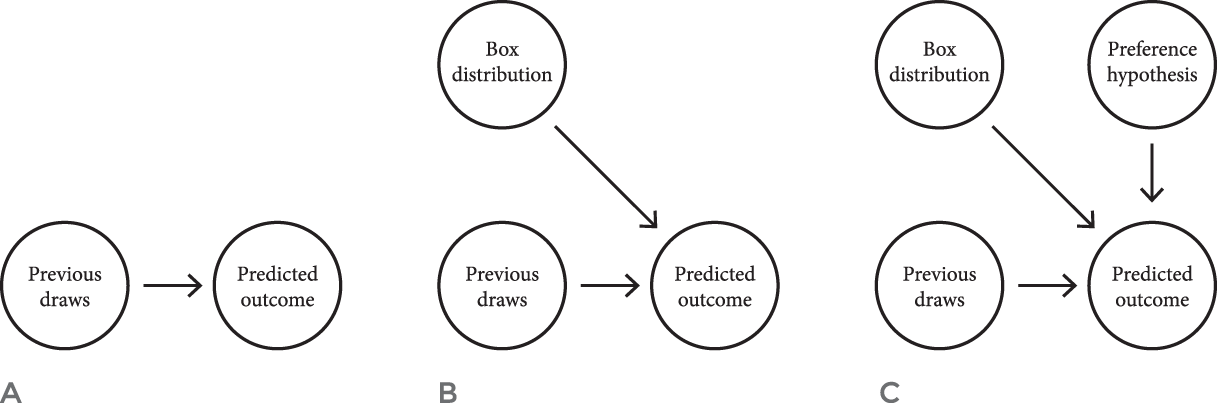


With respect to the conditional probability distributions, we assume that the Box distribution variable represents the statistics of the container, that is, P (majority color) = 0.8 and P (minority color) = 0.2. We ignore the (relatively minimal) changes in the container over time and keep these prior probabilities constant. In the model corresponding with H1, we define P (Pred = C | Prev = C) = 0.8 for both the minority and majority color C; with P (Pred = minority color) = P (Pred = majority color) = 0.5 before any ball is drawn. In H2, we condition on the Box distribution variable as well and have P (Pred = C | Prev = C, Box = C) = 0.8, P (Pred = C | Prev = C, box = ¬C) = 0.2, and P (Pred = C | Box = C) = 1; here, ¬C denotes the opposite color, that is, if C is the minority color, ¬C is the majority color and vice versa. Note that in our model the previous color is always observed and that P (Pred = ¬C) = 1 – P (Pred = C), i.e., this fully defines the conditional probability distribution. In H3, this model still holds for the majority case, but we also condition on the attributed preference as well. We define P (Pred | Prevt = P, Box = B, Hyp = H), where Prevt [with t = 1...4] denotes the t-th ball drawn, as follows:

Pred Box Hyp P(P | B,H) P(P | C_1_,B,H) P(P | C_2_,B,H) P(P | C_3_,B,H) P(P | C_4_,B,H)
C C C 1 1 1 1 1

C C -C 1 1 0.1 0.05 0.01

C -C C 1 1 1 1 1

C -C -C 1 1 0.1 0.05 0.01

Again note that P (Pred = ¬C) = 1 – P(Pred = C) such that this table fully describes the probability distribution for the situation where C1 = C2 = C3 = C4 is the minority color. Given these computational models, we can compute the prediction error for each condition (*Minority*-first/*Majority*-first) and for each of the three hypotheses before every ball drawn. These prediction errors are depicted in Fig 1 in the introduction.

**References**

1. Gweon H, Tenenbaum JB, Schulz LE. Infants consider both the sample and the sampling process in inductive generalization. Proceedings of the National Academy of Sciences. 2010;107(20):9066-71. doi: [10.1073/pnas.1003095107](https://doi.org/10.1073/pnas.1003095107).
2. Jara‐Ettinger J, Sun F, Schulz L, Tenenbaum JB. Sensitivity to the Sampling Process Emerges From the Principle of Efficiency. Cognitive science. 2018;42:270-86. doi: [10.1111/cogs.12596](https://doi-org.browser.cbs.mpg.de/10.1111/cogs.12596).
3. Lucas CG, Griffiths TL, Xu F, Fawcett C, Gopnik A, Kushnir T et al. The child as econometrician: A rational model of preference understanding in children. PloS one. 2014;9(3):e92160. doi: [10.1371/journal.pone.0092160](https://doi.org/10.1371/journal.pone.0092160).
4. Kwisthout J, Bekkering H, Van Rooij I. To be precise, the details don’t matter: on predictive processing, precision, and level of detail of predictions. Brain and cognition. 2017;112:84-91. [doi:10.1016/j.bandc.2016.02.008](http://dx.doi.org/10.1016/j.bandc.2016.02.008).
5. Kullback S, Leibler RA. On information and sufficiency. The annals of mathematical statistics. 1951;22(1):79-86.
6. Clark A. Whatever next? Predictive brains, situated agents, and the future of cognitive science. Behavioral and brain sciences. 2013;36(3):181-204. doi:10.1017/S0140525X12000477.
7. Pearl J. Causal inference without counterfactuals: Comment. Journal of the American Statistical Association. 2000;95(450):428-31.
